# PITYRIASIS VERSICOLOR: A CLINICOMYCOLOGICAL AND EPIDEMIOLOGICAL STUDY FROM A TERTIARY CARE HOSPITAL

**DOI:** 10.4103/0019-5154.44791

**Published:** 2008

**Authors:** Sudip Kumar Ghosh, Sunil Kumar Dey, Indranil Saha, Jayasree Nath Barbhuiya, Arghyaprasun Ghosh, Aloke Kumar Roy

**Affiliations:** 1 *From the Department of Dermatology, R.G. Kar Medical college, Kolkata, India*; 2 *From the Department of Dermatology, M. G. M Medical College, Kishanganj, India*; 3 *From the Department of Community Medicine, R.G. Kar Medical college, Kolkata, India*; 4 *From the School of Tropical Medicine, Kolkata, India*; 5 *From the Department of Dermatology, R.G. Kar Medical college, Kolkata, India*; 6 *From the Department of Dermatology, N.R.S Medical College Kolkata, India*

**Keywords:** *Clinicomycology*, *epidemiology*, *KOH test*, *pityriasis versicolor*

## Abstract

**Background:**

Pityriasis versicolor is a mild, chronic, usually asymptomatic superficial fungal infection of the stratum corneum, caused by Malassezia yeasts. The purpose of the present study is to assess the clinical profile of a group of patients with pityriasis versicolor and to find out the epidemiological characteristics in this part of India as well as any association, if any, with other diseases.

**Materials and Methods:**

For this purpose, 110 consecutive patients of pityriasis versicolor were evaluated clinically and diagnosis was confirmed mycologically at a tertiary care hospital in Kolkata. All data were recorded in a predesigned, pretested semi-structured schedule. The total duration of study period was 12 months.

**Results:**

Majority of the patients were young adults. Most of the patients were asymptomatic. There is prominent seasonal variation of the patients with a peak in August and September months. Most of the lesions were hypopigmented scaly macules and were KOH positive. Most commonly involved sites were chest, face and back. Seborrheic dermatitis sometimes coexisted with pityriasis versicolor and a number of patients also had diabetes mellitus and immunosuppressive conditions.

**Conclusions:**

Overall, the clinicomycological and epidemiological profile of pityriasis versicolor infection as observed in a tertiary care setting in eastern India does not differ significantly from those observed by previous workers elsewhere.

## Introduction

Pityriasis versicolor, also known as tinea versicolor is a superficial chronically recurring fungal infection of the stratum corneum, characterized by scaly, dyspigmented irregular macules most often occurring on the trunk and extremities.^1^ Pityriasis versicolor is caused by Malassezia yeast, a dimorphic fungus. A mycelial phase of the organism predominates in lesions of pityriasis versicolor. According to the taxonomic revision carried out in 1996 on the genus Malassezia, it comprises seven different species.^2^ Malassezia is a member of normal skin flora of human beings. Under certain conditions, the commensal yeast transforms into filamentous pathogenic forms.^3^ It is usually not common in childhood but becomes more common in the late teens with a peak in the early 20s.In tropical countries, the condition is more common than in the temperate zones and as many as 40% of the population may be affected. Although a positive family history is common, genetic relationship is yet to be determined. Clinically, the disease is usually asymptomatic; usually, the patient seeks medical attention for cosmetic blemishes. Cutaneous infection with Malassezia can manifest either as papulosquamous lesions, folliculitis, inverse tinea versicolor or rarely as pityriasis versicolor rubra or erythrasmoid pityriasis versicolor. Wood's lamp examination usually shows yellowish fluorescence of the involved skin.^4^ The organism can readily be identified by treating skin scraping with 10% KOH.^5^ Microscopical visualization of the fungi appears as short, thick hyphae with a large number of variously sized spores (spaghetti and meat-ball appearance). This is diagnostic of pityriasis versicolor.

As it is a member of normal flora of the skin, isolation of Malassezia species from scraping using a lipid rich medium (e.g. olive oil over malt extract or a Tween medium) is of no diagnostic value and is not routinely recommended.

Pityriasis versicolor may be variably associated with various systemic diseases such as diabetes mellitus, Cushing's disease, immunosuppressive conditions and corticosteroid intake. The objective of the present study is to find out the clinical pattern, epidemiological characteristics of pityriasis versicolor as well as any significant associations with other diseases from this part of India.

## Materials and Methods

A descriptive epidemiological study with a cross-sectional design was carried out at Nilratan Sircar Medical College, Kolkata – a tertiary health care set up. The study was carried out over one year and110 consecutive patients of pityriasis versicolor, attending the dermatology OPD, were included for this clinicomycological study. A detailed history regarding the identification of the patient's age, sex, occupation, socioeconomic status (according to the modified Kuppuswamy scale), symptoms, duration, history of recurrence, climatic influence, family history, use of cosmetics, talcum powder, shampoo, oil, synthetic clothing and condition of the personal hygiene were recorded. A thorough clinical examination was done to determine the characteristics and distribution of lesions, color and texture of the skin of the patient and any other associated dermatological or systemic diseases. The cutaneous lesions of pityriasis versicolor were also confirmed by Wood's lamp examination. Mycological confirmation was done by examining KOH-treated skin scraping material under the microscope. Routine laboratory investigations were carried out and screening for HIV infection was done wherever felt necessary. All data were recorded in a predesigned, pretested, semi-structured schedule and analysed accordingly.

## Results

**Fig. 1:**
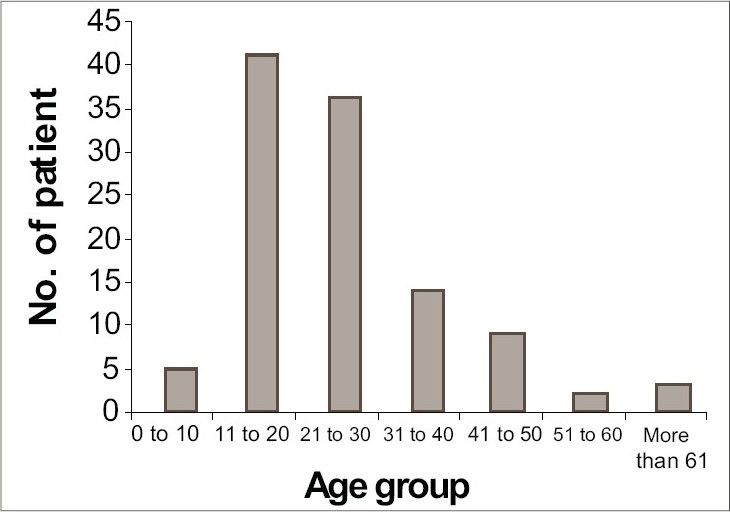
Distribution of pityriasis versicolor patients according to age groups

Out of the 110 patients of pityriasis versicolor, 65 (59.09%) patients were male and 45 (40.91%) patients were female, with a slight male preponderance (M:F = 1.44:1). Most of the patients were young adults. Majority 41 (37.27%) were in the age group of 11 to 20 years. The disease was rare above 50 years of age (4.55%) (Fig. 1).

**Fig. 2:**
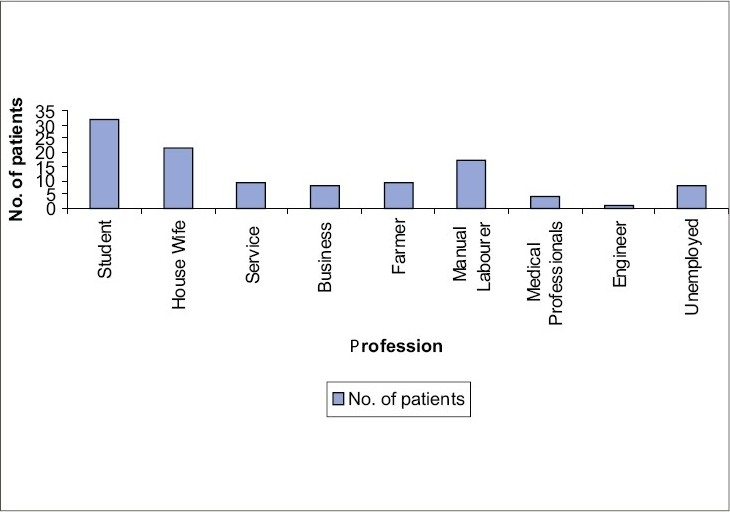
Distribution of pityriasis versicolor patients according to their profession

Fifty percent patients were from upper lower and lower socioeconomic status. Good personal hygiene was noted in 85.45% of patients. Majority of the patients of our study were students (29.09%) (Fig. 2).

**Fig. 3:**
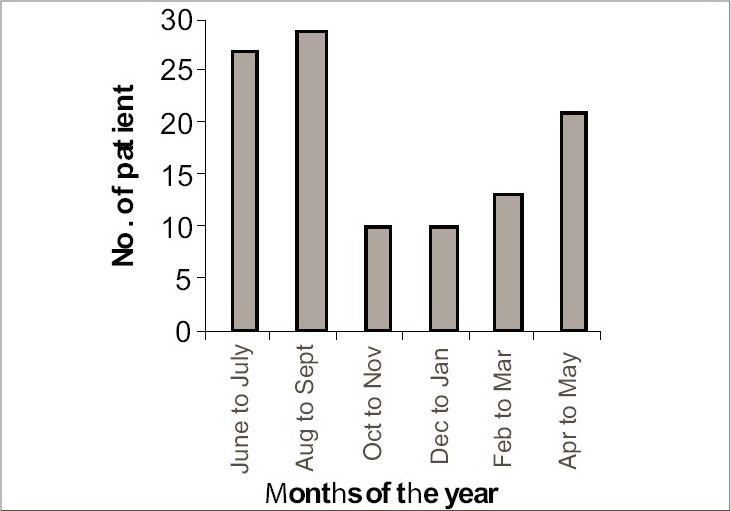
Distribution of pityriasis versicolor patients according to seasonal variations

Most of the patients (52.73%) were asymptomatic, but a large number of patients 47.27% had mild to moderate pruritus. Family history was present in 25.55% of patients. A gross seasonal variation had been noted with a peak incidence during the month of August and September (26.36%) (Fig. 3).

No definite association was found with the use of soap, shampoo, oil, synthetic clothing, talcum powder and cosmetics.

Most of the patients (55.45%) had medium skin complexion with normal (75.45%) skin texture. Most of the lesions were seen over the chest (48.18%) followed by face (46.36%) and back (41.82%).

**Fig. 4:**
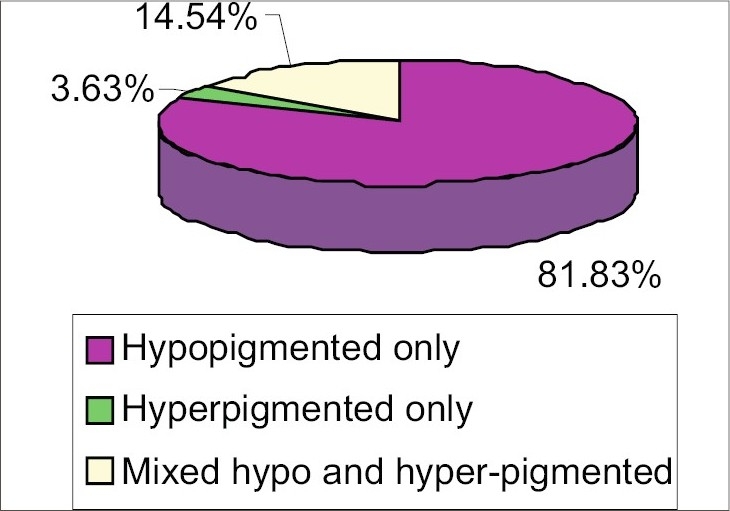
Distribution of pityriasis versicolor patients according to the color of the lesions

Flexural lesions were found rather uncommonly (4.55%). Morphologically most lesions were hypopigmented macules (81.83%) (Fig. 4). Most of the patients (54.55%) had duration of their disease ranging from 2 weeks to 2 years, while disease of more than 10 years’ duration was very rare (0.91%). Coexistent seborrheic dermatitis was observed in 11 (10.0%) patients. Amongst the associated systemic conditions, diabetes mellitus (2.73%) and use of systemic steroid or immunosuppressive drugs were the most common (2.73%). Scaling was present in 89.09% of patients, and 83.64% patients were KOH positive. None of the patients were HIV positive.

## Discussion

The present study was done at a tertiary care hospital and it revealed a number of facts, many of which were in conformity with those of previous workers, while some were justified to draw attention for further studies.

In this study, the most affected age group was 11 to 20 years (37.27%), followed by 21 to 30 years (32.73%). A similar finding was also noted by Dutta *et al.*, (age group: 11 to 30 years).,^6^ Rao *et al*., (2002) (age group 21 to 30 years)^7^ and Krishnan *et al.*, (age group: 15 to 29 years).^8^ Similar to our present study, Rao *et al.* and Krishnan *et al.*, also found predominance of male patients. In contrast, the present consensus is that both sexes are equally prone to develop pityriasis versicolor.^7^

In the present study, 50% of the patients of pityriasis versicolor were from upper lower and lower socioeconomic status. Among the patients, 85.45% had good personal hygiene, while 14.55% of the patients lived in unhygienic conditions. Most of the patients were students (29.09%), followed by housewives (20%) and manual laborers (15.46%). Data, particularly in Indian population, is sparse in these aspects, but probably there is no direct association of pityriasis versicolor with poverty or personal hygienic conditions. Since pityriasis versicolor is commoner in young adults, students were found to be the most commonly affected ones in our study. Majority (52.73%) of our study population were asymptomatic, as also reported by Razack AE *et al.*^9^ and Ingordo *et al.*^10^ In our study, 48.18% patients had history of recurrences, which has also been documented by Krishnan *et al.*^8^ Seasonal trend was found in our study as most of the cases presented in the month of August and September. In the study of Dutta *et al.*,^6^ most of the cases presented in the month of July to September and Rao *et al.*,^7^ also revealed clustering of cases (35%) during the summer months.

In the present study, family history was present in approximately 25% of patients. More or less similar results had been obtained by Hafez *et al.*^11^ However, Rao *et al*,^7^ found a bit higher percentage of positive family history (38.30%).

Most of the patients in our study had normal (75.45%) skin texture, followed by oily and dry skin, but data are sparse in this aspect.

Most of the lesions of pityriasis versicolor were hypopigmented, followed by either mixed hypo- and hyperpigmented or only hyperpigmented. Almost similar observation was done by Krishnan *et al.*,^8^ (84% hypopigmented, 9% hyperpigmented, 6% mixed and 1% erythematous). Rao *et al.*, also showed predominance of hypopigmented lesions (75%).^7^ But this result does not corroborate with that of Aljabre *et al.*,^12^ who concluded that tinea versicolor does not tend to be significantly hypopigmented in dark skinned individuals. This variation may probably be due to differences of climates in different study populations. In the present study, common sites of affection were chest, face and back, but flexural involvement was rare. Similar findings were also noted by Dutta *et al.*^6^ Rao *et al.*^7^ and Krishnan *et al.*,^8^ revealed upper trunk, neck and face to be the commonly involved sites. In contrast, Aljabre *et al.*,^13^ showed that flexural lesions of pityriasis versicolor were not uncommon. Macules were most common lesions found in our study, as also shown by other workers.^7^,^8^ Ten percent of patients of our study had coexisting seborrheic dermatitis. Similar coexistence (11.60%) was also noted by other workers.^8^ In the present study, a small number of patients with pityriasis versicolor had coexisting systemic diseases such as diabetes mellitus (2.73%) and lymphoproliferative malignancies (1%). Some of them were on systemic steroids or other immunosuppressive drugs (2.73%), and 1.82% were pregnant. But studies of some other workers^8^ revealed no association with diabetes mellitus, corticosteroid therapy or other immunosuppressive disorders. In contrast to our study, Rao *et al.*,^7^ showed relatively lower rate of presence of scaling (75%) and positive KOH test (46.60%) as compared to 89.09% and 83.64%, respectively in our study. We conclude that the overall clinicomycological and epidemiological profile of pityriasis versicolor infection in eastern India as observed in a tertiary care hospital does not differ significantly from those observed by previous workers.
